# Determinants of primary care physicians’ intention to provide breast cancer screening services for rural women: a structural equation model based on the theory of planned behavior

**DOI:** 10.3389/fpubh.2025.1674081

**Published:** 2025-11-06

**Authors:** Yinren Zhao, Zixuan Zhang, Yubai He, Zixin Gu, Fan Yang, Zhiqing Hu, Yuan He

**Affiliations:** 1 School of Law and Public Administration, Nanjing University of Information Science & Technology, Nanjing, China; 2 Institute of Medical Humanities, Nanjing Medical University, Nanjing, China; 3 School of Marxism, Nanjing Medical University, Nanjing, China; 4 School of Health Policy & Management, Nanjing Medical University, Nanjing, China; 5 School of Education Science, Jiangsu Normal University, Xuzhou, China; 6 School of Public Health, Nanjing Medical University, Nanjing, China; 7 Laboratory for Digital Intelligence & Health Governance, Nanjing Medical University, Nanjing, China

**Keywords:** breast cancer, primary care physicians, theory of planned behavior (TPB), intention, structural equation modeling

## Abstract

**Background:**

Breast cancer has been a serious health problem worldwide. Early detection is undoubtedly effective in combating severe public health problems in developing countries. Meanwhile, primary care physicians play an important role in implementing screening programs. The objective of our study was to evaluate the determinants of primary care physicians’ intention to provide the breast cancer screening services (BCSs) for rural women.

**Methods:**

We conducted a cross-sectional survey in 24 towns in Jiangsu Province. A total of 1,101 primary care physicians participated in and completed the study. The data collection tool was developed based on the theory of planned behavior (TPB), which includes attitude, subjective norms, and perceived behavioral control, as well as extended components including knowledge of BCSs and past providing-BCSs behavior.

**Results:**

The results of our study showed that subjective norms (*β* = 0.352, *p* < 0.001) had the strongest influence on primary care physicians’ intention to engage in breast cancer screening, followed by attitudes and perceived behavioral control. Both screening knowledge and past screening provision behavior had an indirect effect on behavioral intentions.

**Conclusion:**

The present study demonstrated that extended TPB is an effective model for explaining primary care physicians’ intention to engage in breast cancer screening programs. Meanwhile, our findings provide a reference for governments, hospitals and policies aiming to increase primary care physicians’ intention to provide rural women with BCSs.

## Introduction

Cancer is a major public health, societal, and economic problem in the 21st century, responsible for almost one in six deaths (16.8%) worldwide (1). Breast cancer is the most common and leading cause of death among women (2). According to recent global cancer burden data, breast cancer accounted for 2.3 million new cases in 2022. In China, the largest developing nation, the increase in breast cancer incidence has outpaced the global average since the 1990s, growing at more than twice the global average rate (3). Data from the National Cancer Center (NCC) of China indicate that in 2022, the number of new breast cancer cases among women reached 357,200, making breast cancer the second most common cancer among women (4). The situation is particularly alarming in rural areas, where the age-standardized incidence rate (ASIR) is rising at an annual rate of approximately 6.9%, and the age-standardized mortality rate (ASMR) at about 2.7%, both significantly higher than rates in urban areas (5). These disparities underscore the urgent need for enhanced breast cancer early, diagnosis, and treatment efforts, particularly in rural areas, to address the growing burden and challenges.

Early screening plays a vital role in improving treatment outcomes and prognosis. It not only reduces the incidence and mortality of breast cancer but also enhances the quality of care for women (6). Population-based screening programs have been implemented in many developed countries over the last few decades, leading to significant reductions in mortality and advanced cancer rates (7–9). In China, a national breast cancer screening (BCS) program was launched in 2009 to provide screening services for women aged 35–64. However, despite these efforts, many rural women remain inadequately screened. Chinese epidemiological data indicate that, as of 2019, only 22.3% of women aged 20 years and older and 30.9% of women aged 35–64 had ever undergone breast cancer screening, rates lower than those reported in developed countries (10). Moreover, women with lower socioeconomic status exhibited even lower screening participation. Women aged 20 and above in urban areas had a screening rate of 24.6%, compared to just 15.0% in rural regions (11). The substantial gap between current screening rates and national targets highlights the urgent need to strengthen rural women’s willingness to participate in BCS and to improve their overall health outcomes in China. Primary care physicians, as primary providers of breast cancer screening services (BCSs), play a critical role in improving screening rates. Research suggests that the level of knowledge, attitudes, and encouragement provided by primary care physicians are significant factors in women’s willingness to undergo BCS (12, 13). Additionally, primary care physicians’ recommendations and knowledge also significantly influence rural women’s understanding and willingness to participate in screening programs (14, 15). These studies suggest that exploring primary care physicians’ involvement could be a potential approach to enhancing BCS rates. Previous studies aimed at increasing BCS rates have mainly focused on individuals receiving these services. Most research has examined women’s intentions to participate in BCS (16, 17). Existing studies have explored primary care physicians’ willingness to participate in colorectal, breast, and cervical cancer screening and have identified factors such as knowledge, attitudes, and time constraints as key determinants of provider participation (18, 19). However, few investigations have specifically examined the intentions of primary care providers in rural China to offer breast cancer screening for women. Therefore, this study will apply the theory of planned behavior (TPB) to examine the predictors of primary care physicians’ intentions to provide BCSs for rural women. This focus on providers offers a novel perspective on strategies to enhance screening rates.

TPB, introduced by Ajzen in 1985, is a widely applied social cognitive theory (68). It provides a robust model for predicting and understanding individual behavior across various fields (20, 21). TPB has proven particularly effective in studying healthcare professionals’ behaviors. It has been validated as a useful framework for examining factors that influence physicians’ referral practices and healthcare workers’ job-seeking decisions (22, 23). The theory of planned behavior (TPB) has been widely applied to examine individuals’ breast cancer screening behaviors (24, 25). Building upon this theoretical foundation, the present study employs the TPB framework to investigate the determinants of primary care physicians’ intentions to provide BCSs. The objective of this study is to investigate the determinants of primary care physicians’ intention to offer BCSs and to propose corresponding policy recommendations to strengthen their participation.

### Research model and hypothesis development

**Figure 1:**
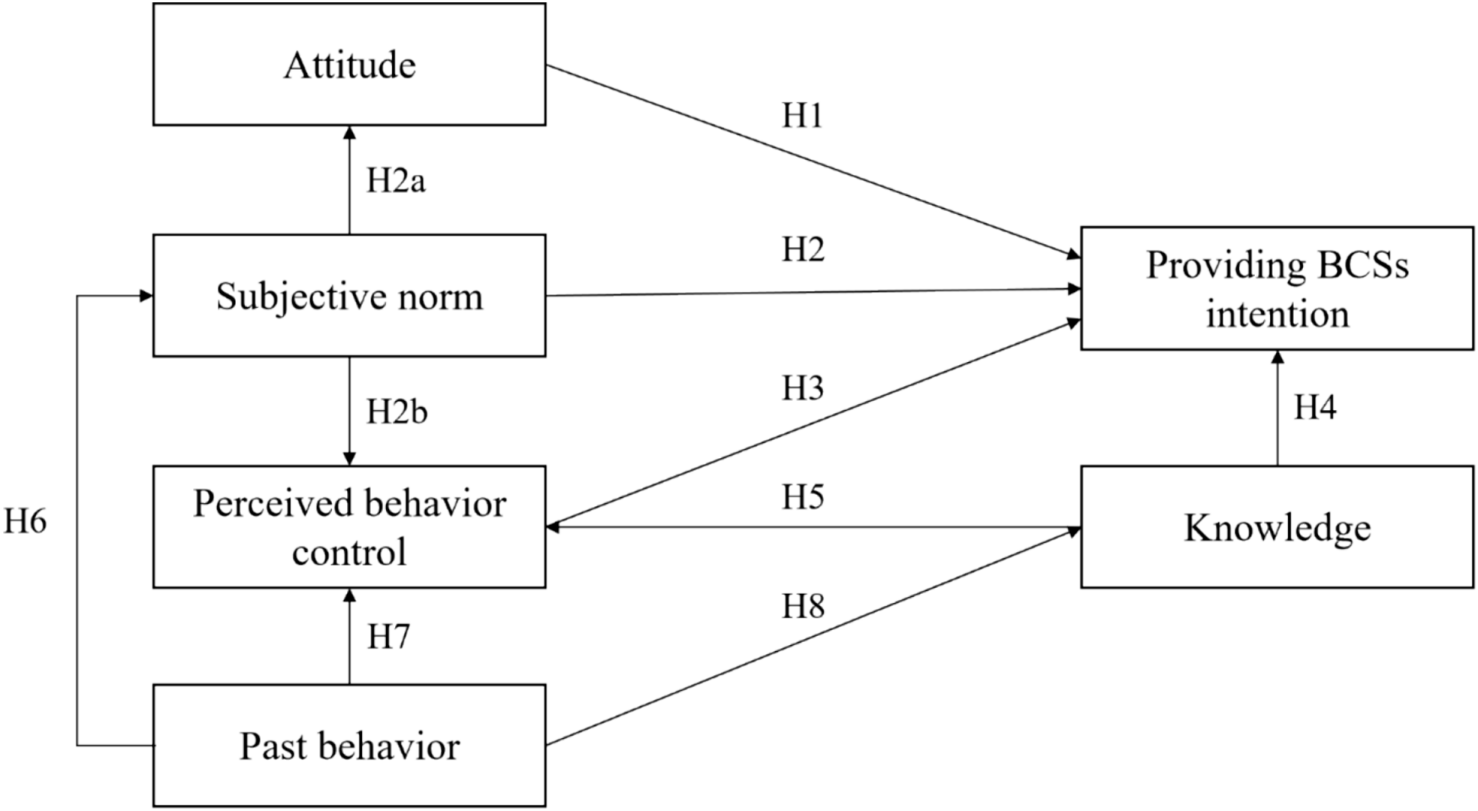
The research model of primary care physicians’ providing BCSs intention.

**Figure 2:**
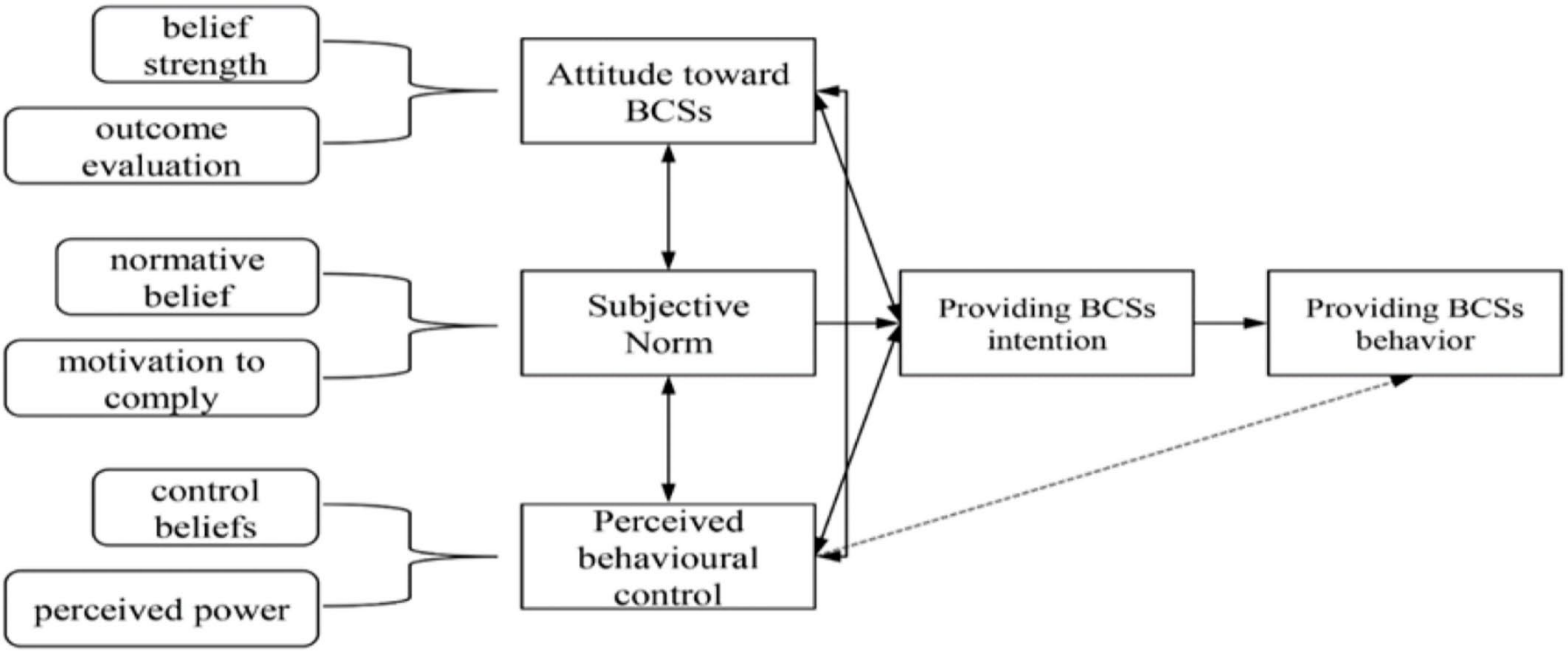
A structural model of the theory of planned behavior.

The current study established a research model (Figure 1) of primary care physicians’ provision of BCS for rural women in China, based on the TPB. TPB is extensively used in predicting and explaining an individual’s behavior under specific conditions. It has demonstrated its effectiveness in anticipating the intentions of various healthcare professionals, including clinicians (26), pharmacists (27), and nurses (28), to provide medical services. In addition, prior meta-analyses and empirical research have indicated that the TPB demonstrates stronger explanatory capacity and clearer conceptual distinctions than alternative psychological models, including the health belief model (HBM) and the theory of reasoned action (TRA) (29, 30). According to TPB, an individual’s intention is determined by three key factors: attitude toward the behavior (A), subjective norms (SN), and perceived behavioral control (PBC) (31, 32). Moreover, SN can affect attitude and PBC, thus indirectly affecting individual’s intentions (see Figure 2).

Attitude refers to an individual’s stable assessment and stance regarding a particular behavior. It can be influenced by strength and belief (*b*) as well as evaluation (*e*). It can be quantified by their multiplication, expressed by the equation: $\mathrm{AB}\propto \sum b_{i}e_{i}$ (where *i* represents the measurement project) (31, 32). Similarly, SN refer to the belief that people will approve of and support a particular behavior, which can be determined by normative beliefs (*n*) and motivation to comply (*m*). The equation is as follows: SN $\propto \sum n_{i}m_{i}$ . Moreover, PBC refers to the extent to which one assesses the difficulty of conducting a specific behavior, which is determined by two distinct factors: control beliefs (*c*) and perceived power (*p*). The corresponding equation is: $\mathrm{PBC}\propto \sum c_{i}p_{i}$ .

In this study, attitude refers to primary care physician’ evaluation of providing BCSs. According to the TPB, A is an important factor that influences an individual’ s intentions and subsequent behavior. Physicians’ intention to provide BCSs is likely to increase if they perceive it as fulfilling personal values, achieving a sense of accomplishment, or recognizing the critical importance of BCS. For instance, Allenbaugh et al. (33) indicated positive attitudes among nurses and residents toward their interactions can significantly enhance the quality of care. Based on this, we propose the following hypothesis:

Hypothesis 1: Attitude is positively correlated with primary physicians’ intention to provide BCSs.

SN refer to the perceived expectations and support of relevant social referents, including colleagues, supervisors, and patients, concerning primary care physicians’ engagement in BCS. Previous study has revealed that SN can directly influence physicians’ intentions to provide BCSs (22, 34). Meanwhile, it can also influence their intentions indirectly by affecting their attitudes and PBC (35, 36). For instance, encouragement and support from hospital leaders, nurses, and colleagues may positively influence physicians’ attitudes toward BCS, alleviate perceived challenges in providing BCSs, and further enhance their motivation. Therefore, we propose the following hypotheses:

Hypothesis 2: SN influences primary physicians' intention positively and directly.

Hypothesis 2a: The more SN primary physicians perceive, the more positive their attitude becomes.

Hypothesis 2b: SN significantly affects the PBC of primary physicians during their attempt to provide BCSs.

In this study, PBC refers to primary care physicians’ perceptions of how easy or difficult it is to provide BCSs, including factors such as the availability of equipment and their own level of technical proficiency. Generally, physicians with greater access to resources and better training tend to feel more confident, enhancing their PBC. According to the TPB, PBC is significantly related to an individual’s intention. Previous research has also shown that PBC effectively predicts physicians’ intentions to provide medical services (34, 37). Consequently, we propose the following hypothesis:

Hypothesis 3: PBC positively and directly influences primary care physicians' intention to provide BCSs.

The predictive utility of the TPB has been shown to improve when additional variables are integrated into the model (38, 39). Additionally, past behavior (PB) and knowledge have been incorporated into the TPB model in health-related studies, supporting their inclusion as variables to enhance the predictive power of TPB (40–42). Among primary care physicians, knowledge of BCS may indirectly influence their behavioral intentions through PBC and attitude. Greater knowledge enhances awareness of the benefits of screening, fostering a more positive attitude toward its provision. Moreover, prior experience with screening helps physicians build procedural familiarity, improving their perceived competence and reducing the perceived difficulty of the task. Such experience may also increase understanding among significant others, thereby enhancing the social support physicians receive when delivering screening services. Consequently, we propose the following hypotheses:

Hypothesis 4: Knowledge of BCS directly and positively influences primary care physicians’ attitudes toward BCSs.

Hypothesis 5: Knowledge of BCS directly and positively influences primary care physicians’ PBC regarding providing BCSs.

Hypothesis 6: Primary care physicians’ PB in providing BCSs influences their SN.

Hypothesis 7: Primary care physicians’ PB in providing BCSs influences their PBC.

Hypothesis 8: Primary care physicians’ PB in providing BCSs influences their knowledge of BCSs.

## Materials and methods

### Participants and data collection

This cross-sectional study was conducted from March 30 to June 1, 2020. A multi-stage stratified cluster sampling method was used to select participants. In the first stage, Jiangsu Province was chosen as the primary sampling unit. Six prefecture-level cities—Lianyungang, Yancheng, Yangzhou, Nanjing, Changzhou, and Wuxi—were selected based on geographic location, socioeconomic status, and distribution of healthcare resources. In the second stage, two rural townships were selected from each city using probability proportional to size (PPS) sampling, resulting in a total of 12 townships. All maternal and child health institutions, community health service centers, health stations, township hospitals, and village clinics within these townships were included as survey sites. In the third stage, primary care physicians at these sites were recruited as study participants. Inclusion criteria required participants to be licensed healthcare professionals with prior experience in providing breast cancer screening (BCS). Exclusion criteria applied to physicians who were ill or otherwise unable to respond. Participants were first recruited through local primary health care centers. Those who consented to take part completed the study in meeting rooms at the respective centers. The research team consisted of trained graduate students. Before the formal experiment, trained investigators distributed paper questionnaires to all participants and explained the study objectives. Upon completing the questionnaire, each participant received a small gift valued at approximately 20 RMB. The required minimum sample size was estimated using Raosoft,^1^ assuming a 95% confidence level, a 5% margin of error, and a 50% response distribution, resulting in a recommended sample size of 384.

### Questionnaire

The development of the questionnaire for this study followed a systematic process to ensure accuracy and validity. Based on our hypothesized model, we referred to existing literature (24, 30), utilized multiple tools to enhance the study, and refined our questionnaire. To ensure accurate representation of theoretical constructs (A, SN, PBC, PB, knowledge and intention) in the instrument, a pilot study was conducted by a panel comprising two experienced quantitative researchers and thirty primary care physicians. The panel reviewed the draft questionnaire and provided feedback to enhance its validity. Based on their suggestions, we revised the wording and phrasing of several items to improve clarity and comprehensibility. The finalized questionnaire comprises two sections:

(1) Demographic and sociological characteristics, including gender, monthly income, and educational background; (2) Intention to provide BCSs, assessed through six subscales. Contained five subscales: attitude toward BCSs, SN, PBC, behavioral intention to provide BCSs, PB and knowledge level of BCSs. The questionnaire was constructed as follows:

#### Attitude subscale

This subscale consisted of behavioral intention (3 items) and related outcome evaluations (3 items). These items measured three key aspects: Primary care providers’ perceived value of the screening work, satisfaction with performing screening tasks, and sense of accomplishment derived from engaging in screening activities. Respondents rated each item on a five-point Likert scale, where higher scores indicated a more positive attitude toward BCSs.

#### Subjective norm subscale

SN was calculated by multiplying the measures of normative beliefs (3 items) and motivation to comply (3 items). Respondents rated the perceived level of support from supervisors, colleagues, and visiting women using a five-point rating scale. Higher scores were strongly correlated with a higher SN.

#### Perceived behavioral control subscale

PBC refers to the perception of hindering or facilitating factors and reflects the personal resources enabling primary care physicians to engage in BCSs. The scale assessed elements such as the effectiveness of hospital equipment, workload, salary, and professional skills. Respondents rated PBC on a five-point Likert scale, where higher scores indicated greater PBC among primary care physicians in performing BCSs.

#### Behavioral intention subscale

Behavioral intention (BI) toward BCSs was measured by three items: “I plan to provide BCSs for rural women” “I am willing to provide BCSs for rural women” and “I try to provide BCSs for rural women.” The response scale ranged from 1 (strongly disagree) to 5 (strongly agree), with higher scores indicating a stronger intention among physicians to participate in BCSs programs.

#### Knowledge subscale

The BCS Knowledge Index was developed to assess primary care physicians’ understanding of BCS. The knowledge scale assessed participants’ understanding of breast cancer and breast cancer screening, including the correct technique for breast self-examination, the appropriate sequence of palpation, and the identification of population groups requiring histopathological examination, among other related topics. It comprised six items, each scored 1 for a correct response and 0 for an incorrect one. The total score, obtained by summing the item scores, ranged from 0 to 6, with higher scores reflecting greater knowledge of BCS among primary care physicians.

#### Past behavior subscale

Four items were used to measure primary care physicians’ past engagement in BCSs. These items evaluated their experience with conducting clinical examinations, performing mammography and ultrasonography, and providing preventive education to rural women. The response scale ranged from 1 (strongly disagree) to 5 (strongly agree), with higher scores reflecting more extensive experience in providing BCSs for rural women.

### Data and statistical analysis

The database was created using EpiData version 3.1. Initially, all data were analyzed using descriptive statistics and exploratory factor analysis in SPSS version 23.0 (IBM Corp., Armonk, NY, USA). The model was constructed using exploratory factor analysis, and correlations between variables were assessed through the Kaiser–Meyer–Olkin (KMO) test and Bartlett’s spherical test. Confirmatory factor analysis (CFA) was subsequently performed using structural equation modeling (SEM) to assess the model’s reliability and stability. Finally, Amos version 23.0 (IBM SPSS Amos, Armonk, NY, USA) was used to fit the hypothetical research model and analyze relationships between variables through the maximum likelihood estimation (MLE) method, with statistical significance set at *p* < 0.05.

### Ethics approval

The study was conducted in accordance with the Declaration of Helsinki and approved by the Ethics Committee of Sir Run Run Hospital, Nanjing Medical University (Protocol code: 2019-SR-017; approved on August 19, 2019). Informed consent details were provided on the first page of the questionnaire. Participants completed the questionnaire after giving informed consent. All responses were collected anonymously.

## Results

### Sample characteristics

A total of 1,205 questionnaires were distributed, and 1,101 valid responses were collected, resulting in a valid response rate of 91.37%. Questionnaires with extreme data were excluded. According to the recommendation by Bagozzi and Yi (43), the sample size meets the requirements for subsequent structural equation modeling analysis. The sample composition is shown in Table 1. The majority of respondents were female (90.7%), whereas only 9.3% were men. Most primary healthcare providers reported relatively low-income levels, with only 12.4% earning more than 8,000 RMB per month. In terms of educational attainment, the majority (62.5%) held a bachelor’s degree. A substantial proportion (79.8%) were employed at community health service centers or township health centers.

**Table 1:** Demographics and relevant characteristics of participants.

Demographic variables	Frequency (N)	Percentage (%)
Gender		
Male	102	9.3
Female	999	90.7
Monthly income (RMB)		
≤3,000	112	10.2
3,000—5,000	373	33.9
5,000—8,000	479	43.5
≥8,000	137	12.4
Level of education		
Master and doctor	27	2.5
Bachelor	688	62.5
Associate degree	297	27
Others	89	8.1
Type of hospital		
Township health center	431	39.1
Village clinic	76	6.9
Rural community health center	448	40.7
Rural maternal and child health center	91	8.3
Other	55	5

### Descriptive analysis

The results of the descriptive analysis were presented in Table 2. Primary care physicians in this study demonstrated a generally favorable attitude toward providing BCSs to rural women, the average mean score of variable attitude was 18.79 ± 4.53 (range: 5–25). However, participants reported limited support from colleagues, patients, and leaders during their daily practice of BCSs (mean = 17.00, SD = 3.94, score ranging from 5 to 25). Moreover, their PBC over engaging in BCSs was relatively low (mean = 11.18, SD = 3.76, score ranging from 5 to 25). Despite these challenges, the study revealed that primary care physicians exhibited a relatively high intention to provide BCSs for rural women (mean = 4.07, SD = 0.63, score ranging from 1 to 5) and demonstrated a good knowledge level of BCSs, with a mean score of 3.93 ± 1.28 (range: 0–6). However, their experience in providing BCSs for rural women was relatively limited, with a mean score of 12.43 ± 3.70 (range: 4–20).

**Table 2:** The result of descriptive statistics.

Variable	Mean ± SD	Range
Attitude	18.79 ± 4.53	5–25
Subjective norm	17.00 ± 3.94	5–25
Perceived behavioral control	11.18 ± 3.76	5–25
Intention	4.07 ± 0.63	1–5
Knowledge of BCSs	3.93 ± 1.28	0–6
Past behavior	12.43 ± 3.70	4–20

### Instrument reliability and validity

SPSS 23.0 was employed to perform exploratory factor analysis on the collected data to verify the validity and reliability of the questionnaire. We conducted both exploratory factor analysis (EFA) and confirmatory factor analysis (CFA). The sample was randomly divided into two groups, and half of the original data (*N* = 550) was used to perform the EFA. Factor analysis was first conducted. The Kaiser-Meyer-Olkin (KMO) value was 0.848 > 0.5, the approximate chi-square value of Bartlett’s test of sphericity was 4438.024, and the significance probability was less than 0.001. These results indicate that the data were highly suitable for factor analysis (44). Principal component analysis was applied to extract common factors, and the maximum variance orthogonal rotation method was employed for data analysis. Four common factors with characteristic root exceeding 1 were extracted, with a cumulative contribution rate of 77%. The factor loadings of individual items ranged from 0.752 to 0.867. Cronbach’s alpha coefficients for all four dimensions were above 0.8, indicating high internal reliability.

Confirmatory factor analysis (CFA) was conducted on the remaining half of the data (*n* = 551) using AMOS 23.0 to assess structural, convergent, and discriminant validity. The maximum likelihood method was applied to assess the model fit. The results indicated that the *χ*^2^/df ratio was 2.211 (<5), RMSEA (root mean square error of approximation) was 0.047 (<0.050), and SRMR (standardized root mean square residual) was 0.034 (<0.08). Additionally, CFI (comparative fit index), TLI (Tucker-Lewis index), and NFI (normed fit index) values all exceeded 0.90, demonstrating strong structural validity and a satisfactory model fit (45). As shown in Table 3, the standardized path coefficients for the four latent variables were all above 0.5. The average variance extracted (AVE) for each latent variable exceeded 0.5 (46), and the combined reliability surpassed 0.7 (47), indicating that the convergent validity is acceptable. Discriminant validity was assessed using the Fornell-Larcker criterion (46, 48). As shown in Table 4, Attitude, SN, PBC, and BI were significantly correlated (*p* < 0.001), yet the correlations between constructs were all lower than the square root of their respective AVEs, indicating adequate discriminant validity. This finding indicates that the variables were closely interrelated yet distinct from one another. Therefore, the scale demonstrated satisfactory discriminant validity (49).

**Table 3:** Convergent validity test (*n* = 550).

Variables			Factor loading	CR	AVE
Attitude	←	A1	0.901	0.892	0.735
	←	A2	0.933		
	←	A3	0.724		
SN	←	SN1	0.761	0.884	0.656
	←	SN2	0.896		
	←	SN3	0.848		
PBC	←	PBC1	0.848	0.852	0.596
	←	PBC2	0.587		
	←	PBC3	0.909		
	←	PBC4	0.784		
BI	←	BI1	0.774	0.824	0.664
	←	BI2	0.800		
	←	BI3	0.911		

A, attitude; SN, subjective norm; PBC, perceived behavior control; BI, behavior intention; CR, combined reliability; AVE average variance extracted.

**Table 4:** Discriminant validity test (*n* = 551).

Variable	Attitude	SN	PBC	BI
Attitude	**0.857**			
SN	0.591^***^	**0.810**		
PBC	0.284^***^	0.375^***^	**0.772**	
BI	0.494^***^	0.546^***^	0.348^***^	**0.815**

SN, subjective norm; PBC, perceived behavioral control; BI, behavior intention; Diagonals (in bold) represent the square root of the AVE. ***, *p*< 0.001.

### Test of structural equation model

**Figure 3:**
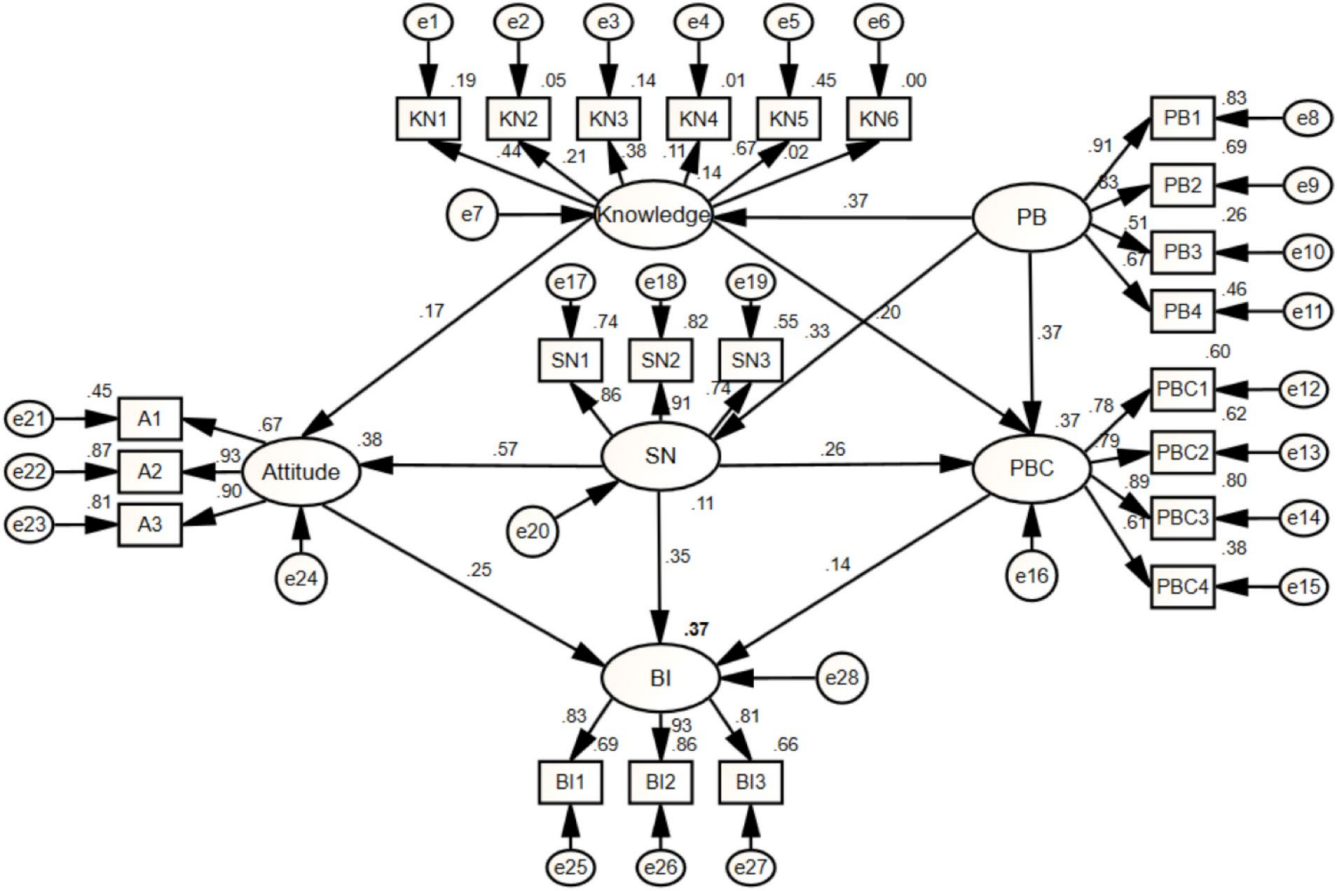
The results of SEM analysis. BI, behavior intention; SN, subjective norm; PBC, perceived behavior control; PB, past behavior.

In this study, the extended structural equation model included knowledge and PB as additional predictors of primary care physicians’ breast cancer screening behavior. Model fit and path analyses were performed using AMOS software. As shown in Table 5, the goodness-of-fit indices indicated that the model achieved a satisfactory fit. The standardized path coefficients between latent variables are presented in Figure 3, while the hypothesis testing results are summarized in Table 6, the relationships among the variables in the structural equation model are all statistically significant (*p* < 0.001). Thus, all ten hypotheses were supported, indicating that the relationships among the variables were statistically validated. As indicated by the results, attitudes towards BCS (*β* = 0.251, *p* < 0.001) and PBC (*β* = 0.140, *p* < 0.001) were significantly associated with primary physicians’ intention to provide BCSs. SN were strongly associated with attitudes among primary care physicians (*β* = 0.573, *p* < 0.001) and moderately associated with PBC (*β* = 0.258, *p* < 0.001) and BI (*β* = 0.352, *p* < 0.001). Additionally, knowledge about BCS significantly influenced PBC (*β* = 0.204, *p* < 0.001) and attitudes (*β* = 0.168, *p* < 0.001) among primary care physicians. However, past screening behavior had moderate effects on SN (*β* = 0.326, *p* < 0.001) and PBC (*β* = 0.370, *p* < 0.001) and knowledge (*β* = 0.368, *p* < 0.001).

**Table 5:** Results of structural equation modeling analysis (*n* = 1,101).

*χ*^2^/df	CFI	TLI	NFI	RMSEA	SRMR
2.618	0.969	0.964	0.950	0.038	0.0411

*χ*^2^/df, relative chi-square and degrees of freedom; GFI, goodness of fit index; TLI, Tucker-Lewis index; NFI, normative fit index; RMSEA, root mean square error of approximation; SRMR, standardized root mean square residual.

**Table 6:** Results of structural equation modeling analysis.

The hypothesis (H)		S.E.	C.R.	Estimate	P	Supported
H1:	BI ← attitude	0.031	0.004	0.251	***	Yes
H2:	BI←SN	0.053	0.006	0.352	***	Yes
H2a:	Attitude ← SN	0.704	0.038	0.573	***	Yes
H2b:	PBC ← SN	0.258	0.031	0.258	***	Yes
H3:	BI←PBC	0.021	0.005	0.140	***	Yes
H4:	Attitude ← knowledge	6.197	1.379	0.168	***	Yes
H5:	PBC ← knowledge	6.148	1.322	0.204	***	Yes
H6:	SN ← PB	1.233	0.125	0.326	***	Yes
H7:	PBC ← PB	1.399	0.138	0.370	***	Yes
H8:	Knowledge ← PB	0.046	0.007	0.368	***	Yes

BI, behavior intention; SN, subjective norm; PBC, perceived behavior control; PB, past behavior.

## Discussion

This study examined the multifaceted predictors of primary care physicians’ intention of providing BCSs to rural women. To our knowledge, this is the first study to examine physicians’ BCS behaviors using the TPB as a conceptual framework. Notably, 90.7% of participants were female, a pattern that aligns with the gender composition of rural primary healthcare personnel in China and the breast cancer screening context (50). In general, the results showed that primary care physicians held high intentions to provide BCSs. The findings of the present study align closely with those of a previous investigation conducted in Tianjin, China, which reported that approximately two-thirds of physicians were willing to engage in cancer screening (18). The high intention demonstrated by primary care physicians in this study may be attributed to a national BCS program launched in 2009 to provide screening services for women (51). This program highlights the importance of promoting BCS and reflects the government’s commitment to its widespread implementation. Such emphasis may increase primary care physicians’ willingness to provide screening services. The expanded TPB model accounted for 37% of the variance in consumption intentions, aligning with results reported in previous studies (52, 53). In accordance with the hypotheses based on the TPB, the results of the path analysis test ascertained that primary care physicians’ attitude, SN, PBC, PB and knowledge level can all directly or indirectly affect their intention to perform the BCSs. These findings confirm suggestions that the TPB is a suitable theoretical basis for understanding physicians’ intentions and behaviors (54–56).

In this study, attitude was found to have a positive impact on the BI of primary care physicians, this finding indicates that physicians’ awareness of the significance of BCS, combined with a sense of fulfillment and professional value derived from delivering such services, is positively associated with their intention to engage in this behavior. This conclusion aligns with research by Heena et al. (12) and Malve et al. (57). In this study, primary care physicians demonstrated positive attitudes toward BCS, suggesting broad acceptance of screening services within China’s primary care system. Additionally, we found that screening-related knowledge indirectly influenced physicians’ intention to offer services through its impact on attitudes, this indicates that improving knowledge may help providers better understand the importance of BCS, thereby fostering stronger support for its implementation. A previous study indicates that in resource-limited settings, physicians’ knowledge of tele-surgery is significantly associated with their attitudes toward its use (58). Furthermore, our findings suggest that external support—from colleagues or supervisors—can enhance service willingness by shaping more favorable attitudes, this may be because support from significant others helps create a more favorable work environment, thereby enhancing primary care providers’ perceived value of participating in screening programs. For physicians with negative attitudes, developing deeply tailored messages to address their specific concerns can effectively help reshape their perspectives (38). For example, emphasizing that BCS is a priority in national health initiatives, highlighting the significant demand for these services among women, and underscoring the scarcity of providers can all serve to counteract negative attitudes and foster a more positive attitude.

In this study, SN has the most significant impact on the intention of primary physicians to provide BCSs. This suggests that physicians who receive support from their supervisors, colleagues, and patients are more likely to have a stronger intention to perform screening activities. Moreover, SN indirectly influenced BI through attitudes and PBC. These findings were consistent with previous studies, a study examining BI among family medicine residents identified SN as the strongest predictor of intention (*β* = 0.56, *p* < 0.001) (59). Another study demonstrated that conformity to colleagues’ practices or expectations significantly predicted healthcare providers’ intentions to perform vaginal examinations for female patients (60). Among all variable, SN2 (support from visiting women) had the highest factor loading (0.906), indicating that support from patients is the most influential factor in motivating primary care physicians, this finding underscores the importance of enhancing rural women’s recognition of primary care physicians as a method to strengthen physicians’ intentions to provide BCSs. In Chinese cultural contexts, rural women often hold more conservative attitudes toward their bodies and are therefore more likely to experience embarrassment or feelings of shame when undergoing physical examinations by others (25, 53). Discrete choice experiment result has also indicated that women prefer screening services provided by primary healthcare workers with a more positive attitude (61). Therefore, fostering a positive doctor-patient relationship and enhancing rural women’s support for BCS conducted by primary healthcare providers may be an important way to strengthen these providers’ willingness to offer such services. A potentially feasible strategy is to disseminate accurate information about BCS to rural women, ensuring they have a comprehensive understanding of the basic procedures and what to expect during the screening process. This knowledge can help alleviate fear stemming from uncertainty. Moreover, hospitals should create a supportive environment, enhance the communication skills of primary healthcare providers, and build a trustworthy image of BCSs providers. Rural women who receive high-quality BCSs are more likely to support BCSs providers, thereby encouraging primary care physicians to provide these services with stronger intention and greater quality. Such reciprocal dynamics can create a virtuous cycle: support and encouragement from rural women increase primary care physicians’ SN and strengthen their intention to provide BCSs. In turn, these physicians are more capable of providing high-quality BCSs, further reinforcing rural women’s trust and support.

According to the TPB, PBC is a key predictor of an individual’s intention to perform a given behavior (31). In this study, participants reported the lowest scores on the PBC dimension, suggesting that primary care physicians in rural areas face considerable challenges in providing BCSs. These challenges include heavy workloads, insufficient equipment, and limited technical skills. Such practical barriers significantly undermine physicians’ intention to engage in BCSs. The significant disparities in financial resources and healthcare infrastructure between urban and rural regions in China have created persistent challenges for rural primary care facilities. Experienced and skilled physicians often prefer working in urban areas with better infrastructure and resources. In contrast, rural primary care facilities, which typically lack adequate support and opportunities, struggle to retain such professionals (62). Meanwhile, studies have indicated that rural primary healthcare institutions in China are often understaffed and lack essential screening equipment such as ultrasound and mammography devices. Only a small proportion are equipped to perform ultrasound imaging, mammography, HPV testing, and liquid-based cytology (63, 64). These findings underscore the pressing need to address systemic issues limiting access to adequate equipment, resources, and skill training in rural areas. The health authority equipping primary care physicians with the skills and resources on BCSs would provide a pathway to improve their BCSs delivery behaviors. The central government should play a key role in balancing healthcare supply and demand by promoting more equitable allocation of medical resources across regions. To reduce barriers faced by primary care providers in delivering BCSs, rural health institutions should focus on both talent recruitment and professional development. On one hand, regular training programs should be provided to primary care physicians involved in screening to strengthen their knowledge and technical capacity (65). On the other hand, policies aimed at attracting and retaining skilled medical professionals in rural areas are essential (66). These include creating clear career advancement pathways, supporting continuing education, increasing investment in screening equipment, and improving government coordination and institutional support for screening programs at all levels of the rural healthcare system.

Our study also revealed that PB, as an external variable, indirectly influenced primary care physicians’ intentions to provide BCSs through its effects on knowledge, SN, and PBC, this finding aligns with some previous studies. Shi et al. incorporated past related health behaviors into the TPB model and found that elementary school students’ past oral health behaviors were associated with their intention to improve oral health behaviors (67). The overall level of PB reported in our sample was relatively low, suggesting that such screening is not yet routine among primary care providers, particularly in rural areas. These results emphasize the need to strengthen physicians’ screening experience and incorporate habit-forming strategies into policy interventions. Institutions may leverage this insight by creating opportunities for physicians with screening experience to share their knowledge and serve as role models. Therefore, institutions can take advantage of this finding. For instance, primary physicians who have been participating in screening services may set up a special team to regularly publicize their experience. At the same time, more obstetrics and gynecology specialists and general practitioners may be involved in BCS, so as to build a professional team.

### Strength and limitations

One major strength of this study is its relatively large sample size (*n* = 1,101), which enhances the robustness of the findings. Another strength of this study lies in the adoption of a well-established social psychological framework—the TPB. Moreover, the inclusion of knowledge and PB as additional variables further enriched and refined the research model. Finally, unlike earlier research that focused primarily on the demand side, this study adopts a supply-side perspective by examining the factors influencing primary care physicians’ intention to provide BCSs.

Our study has limitations. Firstly, this is a cross-sectional study. As a result, the causal relationship between variables needs to be further verified. Secondly, this research only investigated factors of BI of primary medical staff instead of investigating the specific screening behavior. Although there is a strong correlation between intention and behavior, there remains a need to investigate actual screening service conducted by primary physicians. Moreover, only primary care physicians were studied in this research, many other allied health care professionals can impact screening behaviors. While this ensures a cohort with similar characteristics and measurable this is useful to prove efficacy it would be valuable to further study a more diverse cohort, the samples may be expanded in future studies. Finally, this study included only primary care physicians from Jiangsu Province, which may limit the generalizability of the findings. Future research should recruit physicians from additional provinces to enhance external validity.

## Conclusion

Our study extended the TPB model by incorporating knowledge and PB to predict primary care physicians’ intention to provide BCSs for rural women in China. The study found that attitude, SN, PBC, knowledge and PB significantly influenced physicians’ intentions. These factors should be given greater attention in efforts to improve health outcomes among rural women. Furthermore, targeted interventions that enhance physicians’ knowledge and skills related to BCS, including regular training programs, professional competitions, educational lectures, and other structured activities, may effectively promote physicians’ engagement in BCS initiatives.

## Data Availability

The raw data supporting the conclusions of this article will be made available by the authors, without undue reservation.

## Footnotes

^1^ http://www.raosoft.com/samplesize.html
